# The relative compliance of energy-storing tendons may be due to the helical fibril arrangement of their fascicles

**DOI:** 10.1098/rsif.2017.0261

**Published:** 2017-08-09

**Authors:** Tom Shearer, Chavaunne T. Thorpe, Hazel R. C. Screen

**Affiliations:** 1 School of Mathematics, University of Manchester, Manchester M13 9PL, UK; 2 Department of Comparative Biomedical Sciences, The Royal Veterinary College, Royal College Street, London NW1 0TU, UK; 3 Institute of Bioengineering, School of Engineering and Materials Science, Queen Mary University of London, Mile End Road, London E1 4NS, UK

**Keywords:** collagen, mathematical modelling, micromechanics, nonlinear elastic, structure–function

## Abstract

A nonlinear elastic microstructural model is used to investigate the relationship between structure and function in energy-storing and positional tendons. The model is used to fit mechanical tension test data from the equine common digital extensor tendon (CDET) and superficial digital flexor tendon (SDFT), which are used as archetypes of positional and energy-storing tendons, respectively. The fibril crimp and fascicle helix angles of the two tendon types are used as fitting parameters in the mathematical model to predict their values. The outer fibril crimp angles were predicted to be 15.1° ± 2.3° in the CDET and 15.8° ± 4.1° in the SDFT, and the average crimp angles were predicted to be 10.0° ± 1.5° in the CDET and 10.5° ± 2.7° in the SDFT. The crimp angles were not found to be statistically significantly different between the two tendon types (*p* = 0.572). By contrast, the fascicle helix angles were predicted to be 7.9° ± 9.3° in the CDET and 29.1° ± 10.3° in the SDFT and were found to be statistically highly significantly different between the two tendon types (*p* < 0.001). This supports previous qualitative observations that helical substructures are more likely to be found in energy-storing tendons than in positional tendons and suggests that the relative compliance of energy-storing tendons may be directly caused by these helical substructures.

## 1. Introduction

Tendons have varying mechanical requirements depending on their function. Positional tendons need to be stiff in order to keep joints in place, whereas energy-storing tendons play a role in locomotion [1] and are necessarily more compliant [2]. This specialization of mechanical properties between tendon types occurs despite them being composed of the same elementary materials—primarily collagen type I, which is organized into a hierarchical structure consisting of fibrous subunits of varying diameters, each of which is interspersed with a small amount of predominantly non-collagenous matrix [3]. It is thought that structural and compositional differences in this hierarchy give rise to the differing mechanical properties of different tendon types [4].

**Figure 1.**
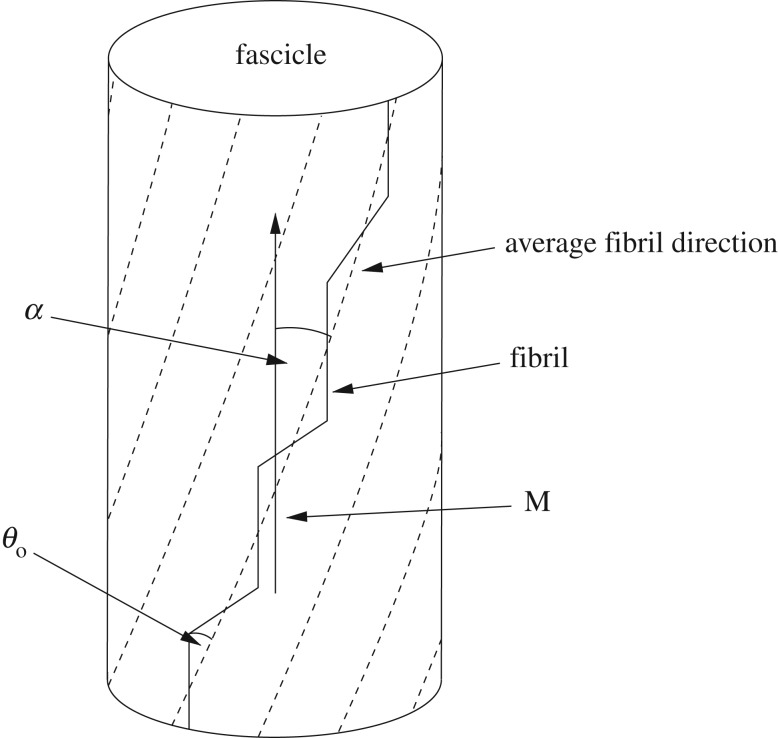
Diagram illustrating the outer fibril crimp angle *θ*_o_, the fascicle helix angle *α* and the fascicle alignment vector **M**. The dashed lines represent the *average* fibril direction upon which the crimp is superimposed.

Mathematical modelling can be used to determine how the geometrical arrangement of tendon subunits affects gross mechanical properties. Many models have been proposed over the last several decades to describe the mechanical behaviour of soft tissues; however, many of these are either phenomenological, or contain a large number of parameters, some of which may be extremely challenging to measure experimentally. Recently, however, two models were developed [5,6] with the aim of having a microstructural basis, while keeping the number of parameters to a minimum. The latter of these requires only two constitutive parameters and four structural quantities, namely: the collagen fibril Young's modulus *E*, the matrix shear modulus *μ*, the collagen volume fraction *ϕ*, the outer fibril crimp angle *θ*_o_, the fascicle helix angle *α* (this term was referred to as the *fibril* helix angle in [6]) and the fascicle alignment vector **M** (figure 1). All of these quantities can potentially be measured via either mechanical testing [7], histology [8], polarized light microscopy [9] or X-ray micro-computed tomography [10–12].

In this report, our goal is to take this recently published model [6] and use it to assist in the interpretation of previous mechanical testing data, comparing the stress–strain behaviour of two types of equine tendon: one positional—the common digital extensor tendon (CDET), and one energy-storing—the superficial digital flexor tendon (SDFT). Taking this approach, we have shown that the differences in mechanical properties between the two tendon types can be entirely explained as arising from differences in the geometrical arrangement of collagen within the fascicles, and not from differences in their constitutive parameters.

## 2. Material and methods

### 2.1. Mechanical testing

The mechanical test data were collected for a previous study [13] and the testing protocol is described in detail therein. Briefly, the CDET and SDFT were dissected from the left forelimbs of 18 horses aged 3–20 years and frozen until the day of testing. On the day of testing, tendons were thawed at room temperature and their cross-sectional areas were measured at the mid-metacarpal level using an alginate paste casting technique that has been shown to be accurate to within 0.8% [14]. The tendons were mounted vertically in a servo-hydraulic materials testing machine (Dartec Ltd, Stroubridge, UK) with a 50 kN load cell and were gripped with cryoclamps cooled by liquid carbon dioxide [15]. They were pre-loaded to 25 N (CDET) or 100 N (SDFT) and were subjected to 20 preconditioning cycles between 0 and 5.25% strain at a frequency of 0.5 Hz, using a protocol adapted from [16]. The load was then removed so that slack was visible in the tendons, which were then tested to failure at a rate of 5% s^−1^. The start point of the test was taken as the displacement at which the initial pre-load was reached (prior to preconditioning). The stresses in the tendons were recorded as forces per unit undeformed areas, so that the reported values are nominal stresses, and stress–strain curves were plotted for each tendon.

### 2.2. Mathematical modelling

Each tendon is modelled as an incompressible, transversely isotropic, nonlinear elastic cylinder, subjected to a longitudinal stretch λ (≥1), so that the deformation gradient is given by [6]2.1
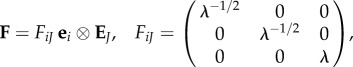
where **e**_*i*_, *i* = (*r*, *θ*, *z*), and **E**_*J*_, *J* = (*R*, *Θ*, *Z*), are deformed and undeformed unit vectors in the radial, azimuthal and longitudinal directions, respectively. The longitudinal stretch is related to the longitudinal strain *e* via λ = 1 + *e*. To calculate the theoretical nominal stresses, the strain energy function from [6] is used:2.2
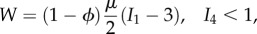
2.3
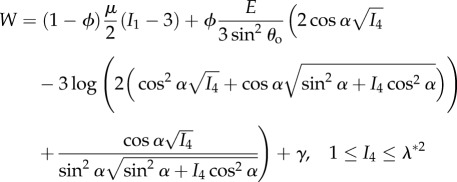
and2.4
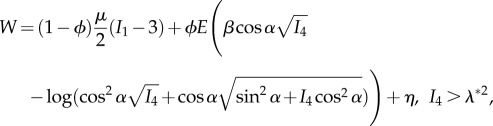
where *I*_1_ and *I*_4_ are strain invariants as defined in [17], for example, 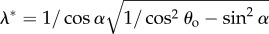 is the critical stretch at which the toe region ends [6], *β* = 2(1 − cos^3^*θ*_o_)/(3 sin^2^*θ*_o_),2.5
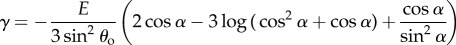
and2.6
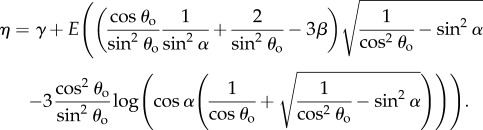
Equations (2.1)–(2.3) can be substituted into the general equation for the nominal stress in a transversely isotropic nonlinear elastic material, which, for a strain energy function that is only dependent on *I*_1_ and *I*_4_, is given by2.7
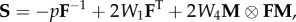
where *p* is a Lagrange multiplier associated with the incompressibility constraint, *W*_*i*_ = ∂*W*/∂*I*_*i*_ and **M** is a unit vector oriented in the direction of the fascicles in the undeformed configuration. It is assumed that the fascicles are coaligned with the longitudinal axis of the tendon in both the CDET and SDFT, so that **M** = **E**_*Z*_. In reality, this is not the case; however, it is assumed that the deviation from longitudinal alignment is small enough to be negligible.

Upon applying stress-free boundary conditions on the curved surface of the cylinder, thus determining the value of *p*, the following expression is obtained for the longitudinal nominal stress:2.8
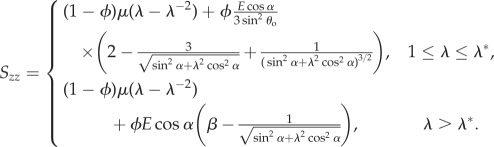
This expression was used to model the mechanical test data obtained for the CDET and SDFT.

### 2.3. Parameter selection

#### 2.3.1. Constitutive parameters

For the collagen Young's modulus, there is a wide range of reported values in the literature, ranging from 32 MPa [18] to 16 GPa [19]. To the authors' knowledge, there are no data available for equine collagen fibrils; therefore, bovine data were used as a substitute—the value selected here was 1.9 GPa, which is the value reported by Grant *et al.* [7] for bovine collagen fibrils under ambient conditions.

There is a lack of data in the literature for the matrix shear modulus due to the difficulties involved in measuring it experimentally; therefore, a custom method was developed to estimate the values of this parameter in the CDET and SDFT based on mechanical test data from a previous study [13]. The testing protocol is described in detail within that paper; however, briefly, groups of two fascicles bound together by the interfascicular matrix were dissected from the CDET and SDFT (*n* = 17, 12 samples per tendon). The fascicles were secured into a custom-made dissection rig and the opposing end of each fascicle was cut transversely, leaving 10 mm of intact interfascicular matrix. The intact end of each fascicle was then secured in a materials testing machine and pulled apart to failure at a speed of 1 mm s^−1^. Force and extension data were recorded adopting the point at which the load reached 0.02 N as the test start point. The matrix shear modulus was then estimated using the following equation:2.9
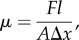
where *F* is the force and Δ*x* is the extension in the matrix at 10% of the failure load, *l* is its thickness and *A* is its contact area. The contact area was estimated by multiplying the average fascicle diameter with the test length (10 mm). The thickness was estimated based on values calculated by Ali *et al.* [20]. Using this method, it was estimated that the matrix shear modulus of the CDET is 0.97 kPa and of the SDFT is 1.62 kPa. This was not done on a sample by sample basis, but by using the average values of *F*, *l*, *A* and Δ*x* from the sources described above; therefore, these values should be seen as order-of-magnitude estimates rather than exact quantities. To obtain estimates of bounds for these parameters, this calculation was repeated using the minimum and maximum forces that were recorded for each tendon type. It was found that the CDET shear modulus ranged between 0.16 and 3.08 kPa, and the SDFT shear modulus ranged between 0.32 and 5.52 kPa; therefore, we conclude that the matrix is less stiff than the fibrils by several orders of magnitude.

#### 2.3.2. Structural parameters

For the collagen volume fraction, an estimate was made based on the collagen area fractions reported in [8] for non-incubated rat tail tendon—the selected value was 0.8. The outer fibril crimp and helix angles were used as fitting parameters in order to predict their values. The function (2.7) was used to fit each of the experimental datasets up to 10% strain, beyond which it was assumed that the deformation was no longer elastic. The experimental data were fitted using the *NonlinearModelFit* command in Mathematica 11.0 (Wolfram Research, Inc., Champaign, IL, USA) subject to the constraints 0 ≤ *θ*_o_ ≤ 90° and 0 ≤ *α* ≤ 90°.

The predicted outer fibril crimp angles were used to calculate the *average* crimp angles of each tendon type. The fibril crimp angle has been observed to vary with fascicle radius, being at a minimum in the centre of the fascicle and at a maximum on its periphery in fascicles with longitudinally [3] *and* helically [21] aligned fibrils. Therefore, it is expected that the average crimp angle within a fascicle will be strictly smaller than the outer fibril crimp angle. The model assumes a fibril crimp angle distribution of the following form:2.10
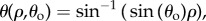
where *ρ* is a non-dimensional variable that ranges between 0 at the centre of a fascicle and 1 on its edge. The average crimp angle was therefore calculated as2.11
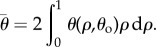


#### 2.3.3. Error analysis

Owing to the nonlinearity in the model, it is possible that some of the parameter sets that were predicted were not globally optimum, but were simply locally optimum based on the initial conditions of the nonlinear solver. It is therefore possible that some of the poorer fits could skew the results. To avoid this problem, any fits with a mean squared error greater than 5 MPa^2^ were removed from the data before they were analysed as described below.

#### 2.3.4. Statistical analysis

To determine whether the predicted crimp and helix angles were statistically different, the *LocationTest* function in Mathematica 11.0 was used, which automatically selects the most appropriate hypothesis test type based on the distribution of the data. In all of the cases considered here, the data were not distributed normally and the Mann–Whitney test was selected with the null hypothesis that the true median difference between the samples being tested was zero and the alternative hypothesis that the difference was not zero.

#### 2.3.5. Sensitivity analysis

As mentioned above, there is an extremely wide range of values reported for the collagen Young's modulus in the literature. To investigate the sensitivity of the model's predictions to the assumed constitutive parameters, the fitting process described above was repeated using the following values for the collagen Young's modulus: 30 MPa, 100 MPa, 1 GPa, 3 GPa, 10 GPa, 20 GPa, and the following values for the collagen volume fraction: 0.4, 0.6, 1.0, in addition to the original values of *E* = 1.9 GPa (§2.3.1) and *ϕ* = 0.8 (§2.3.2) in every combination.

## 3. Results

**Figure 2.**
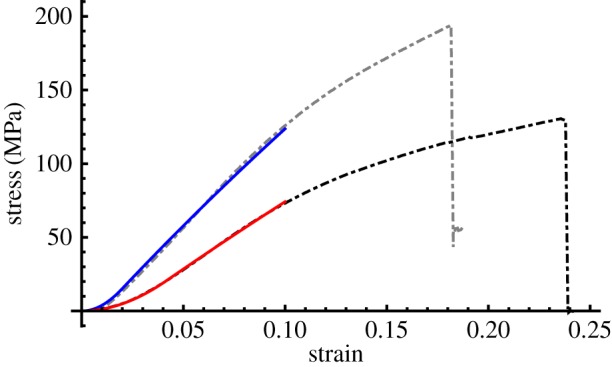
Example experimental (dashed) and theoretical (solid) stress–strain curves for the CDET (grey/blue) and SDFT (black/red). (Online version in colour.)

**Figure 3.**
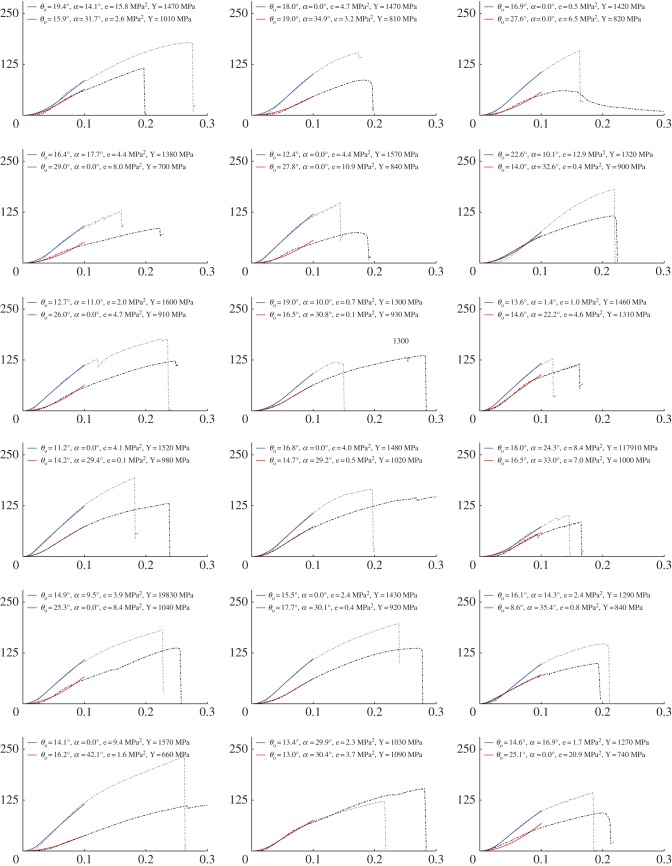
Experimental (dashed) and theoretical (solid) stress (in MPa, vertical axes)–strain (horizontal axes) curves for the CDET (grey/blue) and SDFT (black/red). For each plot, the parameters used to fit the data, *θ*_o_ and *α*, are provided, along with the mean squared error *e* and the maximum tangent modulus *Y* for each sample. (Online version in colour.)

The predicted fibril crimp and fascicle helix angles according to the model fit are listed in table 1 (given as mean ± s.d.) and example fits to the experimental data are plotted in figure 2 (plots of all 36 fits are provided in figure 3). There was no statistically significant difference between the crimp angles of the CDET and SDFT (*p* = 0.572); however, there was a highly statistically significant difference between the helix angles of the CDET and SDFT (*p* < 0.001) according to the Mann–Whitney test.

**Table 1.** Predicted fibril crimp and helix angles.

tendon	outer crimp angle	average crimp angle	helix angle
CDET	15.1° ± 2.3°	10.0° ± 1.5°	7.9° ± 9.3°
SDFT	15.8° ± 4.1°	10.5° ± 2.7°	29.1° ± 10.3°

The results of the sensitivity analysis are reported in table 2. As the table shows, it was only possible to obtain a reasonable number of acceptable fits (defined as at least five for both tendon types) with *E* = 1.9 GPa or 3 GPa and with *ϕ* = 0.6, 0.8 or 1.0, and in all of these cases, there was a statistically highly significant difference (*p* < 0.001) between the predicted helix angles of each tendon type.

**Table 2.** A sensitivity analysis was carried out to determine the effect of the assumed values of the constitutive parameters on the predicted average outer crimp and helix angles. Here, *N*_C_ and *N*_S_ are the number of CDET and SDFT curves, respectively, that were fitted with a mean squared error of less than 5 MPa^2^ in each case. *θ*_C_ and *θ*_S_ are the average predicted outer crimp angles for the CDET and SDFT, respectively. *α*_C_ and *α*_S_ are the average predicted helix angles in the CDET and SDFT, respectively. Every case in which there were enough well-fitted curves to carry out a statistical analysis is indicated with a * and the *p*-values are given where appropriate.

	*E* (MPa)						
*ϕ*	30	100	1000	1900	3000	10 000	20 000
0.4	*N*_C_ = 0	*N*_C_ = 0	*N*_C_ = 0	*N*_C_ = 0	*N*_C_ = 1, *N*_S_ = 15	*N*_C_ = 0, *N*_S_ = 5	*N*_C_ = 0, *N*_S_ = 5
	*N*_S_ = 0	*N*_S_ = 0	*N*_S_ = 0	*N*_S_ = 0	*θ*_C_ = 15°, *θ*_S_ = 16°	*θ*_S_ = 33°	*θ*_S_ = 57°
					*α*_C_ = 30°, *α*_S_ = 23°	*α*_S_ = 28°	*α*_S_ = 28°
0.6	*N*_C_ = 0	*N*_C_ = 0	*N*_C_ = 0, *N*_S_ = 2	*N*_C_ = 1, *N*_S_ = 15	*N*_C_ = 15, *N*_S_ = 10*	*N*_C_ = 0, *N*_S_ = 5	*N*_C_ = 0, *N*_S_ = 4
	*N*_S_ = 0	*N*_S_ = 0	*θ*_S_ = 18°	*θ*_C_ = 15°, *θ*_S_ = 16°	*θ*_C_ = 15°, *θ*_S_ = 17°_*p*=0.647_	*θ*_S_ = 42°	*θ*_S_ = 55°
			*α*_S_ = 21°	*α*_C_ = 30°, *α*_S_ = 22°	*α*_C_ = 7°, *α*_S_ = 29°_*p*<0.001_	*α*_S_ = 28°	*α*_S_ = 34°
0.8	*N*_C_ = 0	*N*_C_ = 0	*N*_C_ = 0, *N*_S_ = 8	*N*_C_ = 14, *N*_S_ = 12*	*N*_C_ = 11, *N*_S_ = 5*	*N*_C_ = 0, *N*_S_ = 5	*N*_C_ = 0, *N*_S_ = 4
	*N*_S_ = 0	*N*_S_ = 0	*θ*_S_ = 16°	*θ*_C_ = 15°, *θ*_S_ = 16°_*p*=0.572_	*θ*_C_ = 13°, *θ*_S_ = 25°_*p*=0.047_	*θ*_S_ = 57°	*θ*_S_ = 56°
			*α*_S_ = 14°	*α*_C_ = 8°, *α*_S_ = 29°_*p*<0.001_	*α*_C_ = 4°, *α*_S_ = 28°_*p*<0.001_	*α*_S_ = 28°	*α*_S_ = 34°
1.0	*N*_C_ = 0	*N*_C_ = 0	*N*_C_ = 1, *N*_S_ = 16	*N*_C_ = 14, *N*_S_ = 8*	*N*_C_ = 3, *N*_S_ = 5*	*N*_C_ = 0, *N*_S_ = 5	*N*_C_ = 0, *N*_S_ = 4
	*N*_S_ = 0	*N*_S_ = 0	*θ*_C_ = 15°, *θ*_S_ = 17°	*θ*_C_ = 14°, *θ*_S_ = 20°_*p*=0.072_	*θ*_C_ = 12°, *θ*_S_ = 28°_*p*=0.091_	*θ*_S_ = 61°	*θ*_S_ = 57°
			*α*_C_ = 30°, *α*_S_ = 20°	*α*_C_ = 6°, *α*_S_ = 28°_*p*<0.001_	*α*_C_ = 0°, *α*_S_ = 28°_*p*=0.029_	*α*_S_ = 28°	*α*_S_ = 34°

## 4. Discussion

The model used above provides a link between the microstructures and mechanical functions of the CDET and SDFT, explaining that the relative compliance of energy-storing tendons may be caused directly by the helical fibril arrangement of their fascicles, and *not* by differences in their fibril Young's modulus or crimp angles. The fitting process predicted outer fibril crimp values similar to those observed experimentally in previous studies (table 3) and in particular, the value predicted for the SDFT (15.8 ± 4.1°) is very close to the range of those for the peripheral SDFT fibrils reported in [23] of 15.9° − 20.1°. The predicted average crimp angles are smaller than the outer crimp angles, as expected, and are of a similar magnitude to the average crimp angles reported in the supplementary material of [4] of 7.0° ± 1.0° in the CDET and 10.2° ± 1.6° in the SDFT.

**Table 3.** Reported values of outer fibril crimp angle.

tendon	crimp angle	methodology	references
rat tail	12.5°–20.0° (age-dependent)	polarized light microscopy	[9]
rat tail	10.7°–27.0° (age-dependent)	theoretical prediction	[9]
rat Achilles	11.84°–14.73° (condition-dependent)	polarized light microscopy	[22]
equine SDFT	15.9°–20.1° (age-dependent)	polarized light microscopy	[23]
rat tail	≈33°	theoretical prediction	[24]
human Achilles	14.7° ± 2.2°	polarized light microscopy	[25]
human biceps brachii	17.3° ± 2.0°	polarized light microscopy	[25]
human quadriceps	16.6° ± 2.0°	polarized light microscopy	[25]
human extensor pollicis longus	12.6° ± 1.5°	polarized light microscopy	[25]
equine CDET	15.1° ± 2.3°	theoretical prediction	this study
equine SDFT	15.8° ± 4.1°	theoretical prediction	this study

A much larger fascicle helix angle in the SDFT than in the CDET was also predicted. This prediction agrees with the qualitative observations in [4], in which rotation was observed in extended SDFT fascicles, but not in CDET fascicles, suggesting the presence of helical substructures in the SDFT, but not in the CDET. This supports the hypothesis that helical substructures are more likely to be found in energy-storing tendons than in positional tendons. Unfortunately, it is not possible to compare the predicted helix angles with experiments *quantitatively* since, to the authors' knowledge, this parameter has not previously been measured.

We note that the CDET has been estimated to experience strains of up to 3% *in vivo* [26] and maximum SDFT strains of up to 16.6% have been measured [27]. Owing to the potential presence of residual strain *in vivo*, it is not possible to directly relate *in vivo* strains to the *ex vivo* strains used in the analysis presented in this paper. In order to make the fitting process fair, it was important for it to take place over the same strain range for each tendon. Our approach was to consider a moderate strain range in between the *in vivo* strains mentioned above. We note that this is possibly beyond the range experienced by the CDET *in vivo*; however, there was no evidence of damage at 10% *ex vivo* strain in the stress–strain curves considered in this paper.

Owing to a lack of data on equine fibrils, bovine data were used for the collagen Young's modulus, and it was assumed that equine collagen is mechanically equivalent to bovine collagen. This may not be the case in reality; however, any mechanical differences are likely to be small as the amino acid sequence in type I collagen is largely conserved between species [28]. Additionally, the sensitivity analysis reported in table 2 revealed that the highest number of good fits was achieved using the values for the collagen Young's modulus and volume fraction that were originally chosen, *E* = 1.9 GPa and *ϕ* = 0.8, which may indicate that these values are close to the true values for equine digital tendons. It was not possible to obtain a reasonable number of good fits with *E* ≤ 1 GPa or *E* ≥ 10 GPa, which suggests that the collagen Young's modulus is of the order of 1 GPa and that some of the values reported in the literature of the order of 10–100 MPa or 10 GPa can be discounted. Similarly, based on this evidence it is reasonable to assume that the true value of the collagen volume fraction is strictly greater than 40%. Owing to the nonlinearity in the model, several different combinations of the helix and crimp angles are always able to achieve a reasonable fit to the experimental data considered here. Therefore, we cannot rule out the possibility that differences in the crimp angles do play a role in the differences in the mechanical behaviour of the two tendons. However, taken alongside the previously mentioned qualitative observations, it is interesting that the best fits tend to be achieved with large differences in the helix angles and small differences in the crimp angles.

It is important to note that both the crimp angle and helix angles contribute to the mechanical properties of tendons. In [6], it was demonstrated that the crimp angle plays a crucial role in the size and shape of the toe region of a tendon's stress–strain curve and that if a tendon had a crimp angle of 0°, it would have no toe region. The crimp angle does not affect the stiffness in the linear region, however, which is probably due to the fact that once this region has been reached, all of the crimp has straightened out and all of the fibrils are taut. The helix angle appears to be able to tune the relative compliance or stiffness of a tendon independently of the crimp angle. Therefore, while modifying the crimp angle is a good way to modulate the length of the toe region and the initial damping in the tendon response, it is not a good way to alter the stiffness of the linear region of the curve or tendon mechanics past the toe region. Having a helical fibril arrangement is a good way round this as this will influence the full length of the stress–strain curve.

## Data accessibility

The mechanical test data are available to download and have the doi:10.17636/01013395.
